# To Die or Not to Die: Cell Death in Biology and Disease

**DOI:** 10.3390/ijms23126734

**Published:** 2022-06-16

**Authors:** Marcus Krüger, Peter Richter

**Affiliations:** 1 Environmental Cell Biology Group, Department of Microgravity and Translational Regenerative Medicine, Otto von Guericke University, 39106 Magdeburg, Germany; 2 Gravitational Biology Group, Department of Biology, Friedrich-Alexander University, 91058 Erlangen, Germany

Cell death is a fundamental and highly organized biological phenomenon that was long considered nothing more than the inevitable endpoint of life; this is reflected in the meaning of the Greek word, ἀπόπτωσις (“falling leaves from a tree”). It took science a long time to realize that the balance between cell proliferation and cell death is a prerequisite for the development and maintenance of multicellular organisms. In 1842, Carl Vogt was the first to describe that the notochord of the midwife toad is removed by cell death during metamorphosis, allowing for the formation of vertebrae [1]. In the 1960s, genetic studies showed that cell death during development depends on the expression of endogenous genes, leading to the concept of programmed cell death [2,3]. Finally, it was electron microscopy that provided ultrastructural evidence for the characteristics of the different types of cell death in the 1970s [4]. Although knowledge of cell death mechanisms has advanced in recent decades and various mechanisms have been described, it remains difficult to reasonably classify different types of cell death because of complex interactions and overlaps between them.

In simple terms, cell death can occur in two ways (Figure 1A). One way is through apoptosis (the programmed cell death or “cell suicide”; today classified as type 1 cell death), a physiological form of cell death that describes the process of active cell death under the control of genes. Cells that die in this manner, e.g., during development or tissue homeostasis, exhibit morphological features that include cytoplasmic shrinkage, nuclear condensation, and the retention of membrane and organelle integrity [4]. There are at least two major signaling pathways that lead to apoptosis: the intrinsic and extrinsic pathways of apoptosis. The extrinsic pathway of apoptosis begins outside of a cell, when conditions in the extracellular environment cause a cell to die. The intrinsic pathway of apoptosis pathway begins when injury occurs inside the cell, and the resulting stress activates the apoptotic pathway [5]. The other major type of cell death is necrosis (type III cell death), an uncontrolled form of cell death that occurs in response to stress events. These cells swell and burst as a result of stressors, such as cell injury, metabolic disturbances, pathogenic invasion, or physiological tissue damage [4]. In addition, two other types of cell death are often distinguished: Autophagy-associated cell death (type II), another type of programmed cell death triggered by self-digestion of cell contents by autophagy (Figure 1B), and entosis (type IV cell death by invasion, Figure 1C).

For this Special Issue, Wilkinson et al. [6] summarized the role of RNA modifications in molecular and cellular stress responses and disease. In response to stresses that can trigger cell death, RNA modifications are mobilized to activate or inhibit signaling pathways that combat oxidative stress, hypoxia, therapeutic stress, metabolic stress, heat shock, DNA damage, or ER stress. However, the role of RNA modifications in the response to these cellular stressors appears to be context- and cell-type-dependent.

It has long been known that epithelial cells, once detached from their extracellular matrix, can undergo a process of apoptotic cell death called anoikis. In 2007, Overholtzer et al. [7] reported that detached epithelial cells can also take an alternative route to death called entosis, which is driven by cell-in-cell invasion (Figure 1C). For this Special Issue, Kianfar et al. [8] summarized the current knowledge of molecular mechanisms and clinical data on entosis, highlighting both conclusive explanations and controversies.

As described above, cell death in the biology of multicellular organisms serves the purpose of removing unwanted or abnormal cells. However, the machinery for cell death is evolutionarily conserved, and elements are also found in unicellular organisms. The molecular mechanisms of cell death (or conversely, cell survival) are complex and often closely linked to other cellular processes, such as cell proliferation or differentiation, and thus they are part of a comprehensive signaling network. The disruption of these mechanisms often leads to developmental defects, and factors that trigger cell death can directly contribute to the pathogenesis of many diseases, including cancer, neurodegenerative diseases, and tissue damage. Apoptosis is tightly controlled by several genes. These genes are highly conserved between species, such as the Bcl-2 family, caspase family, c-Myc, p53 and others [9].

The clearance of apoptotic cells by tissue macrophages and nonprofessional phagocytes is an essential process for maintaining tissue health and function. In particular, the interaction of macrophages with apoptotic cells is fundamental for the efficient regression of inflammation. Knuth et al. [10] found an induction of peritoneal macrophage proliferation in response to apoptotic cells. During inflammation, this could increase macrophage numbers to allow efficient clearance.

Harnessing a cell’s individual death mechanism is a highly effective method and the most successful non-surgical treatment for cancer to date [11]. Targeting apoptosis is also effective in all cancers because the evasion of apoptosis is a hallmark of cancer and is not dependent on the cause or type of cancer. Kiss et al. [12] showed that methyl-donor treatment can reduce tumor cell proliferation rate and promote apoptotic signaling by protecting p53 functions through the downregulation of the MAPK/ERK and AKT pathways in breast and lung adenocarcinoma cell lines. Jozkowiak et al. [13] described for the first time the anticancer effect of DMU-214, gefitinib, and their combination in tongue cancer cells. They indicated that these drugs can induce the intrinsic pathway of apoptosis. Wimmer et al. [14] reported that hypofractionated radiotherapy with 5 × 3.0 Gy was very effective in inducing cell death and modulating immune checkpoint molecules on the cell surface of squamous cell carcinoma of the head and neck (HNSCC), and partially at the mRNA level. The results suggest that a holistic approach is most promising to optimize multimodal treatments for HNSCC, rather than focusing specifically on human papillomavirus (HPV) status, radiotherapy fractionation regimens, or the inhibition of single immune checkpoint molecules.

Taken together, cell death is a complex and controlled cellular process that contributes to development, tissue integrity, and homeostasis in multicellular organisms. Dysregulation leads to various pathological conditions, including cancer. Not surprisingly, the research of Sydney Brenner, H. Robert Horvitz and John Sulstonzur toward identifying genes that control apoptosis was awarded the Nobel Prize in Medicine in 2002. The aim of this Special Issue is to highlight new insights into cell death in disease development and the modulation of cell death to treat disease. In recent years, this field expanded and new mechanisms controlling cell death pathways were uncovered. Researchers are now focusing on harnessing the regulated pathways closely associated with different diseases for diagnosis, treatment, and prognosis. We would like to thank all of the authors who contributed to this Special Issue. Ongoing research offers new insights into the ‘biology of death’ and hope for cures and treatments for a variety of diseases.

## Figures and Tables

**Figure 1:**
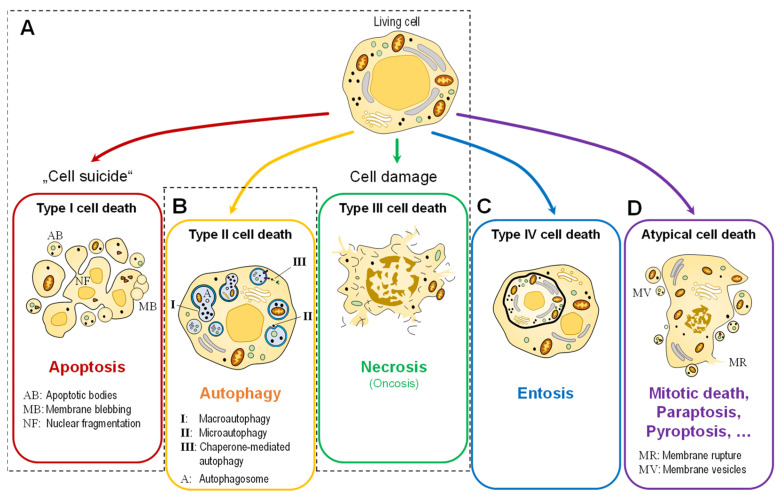
Simplified representation of the morphologically distinguishable types of cell death. (**A**) The two classical main types, apoptosis (type I) and necrosis (type III). Recent research also distinguishes between two further types: (**B**) autophagic cell death (type II) and (**C**) entosis (type IV). (**D**) Other (molecular) types are grouped under the term “atypical cell death”.
